# Additive engineering for efficient wide-bandgap perovskite solar cells with low open-circuit voltage losses

**DOI:** 10.3389/fchem.2024.1441057

**Published:** 2024-09-02

**Authors:** Xixi Yu, Huxue He, Yunuo Hui, Hua Wang, Xing Zhu, Shaoyuan Li, Tao Zhu

**Affiliations:** Faculty of Metallurgical and Energy Engineering, Kunming University of Science and Technology, Kunming, China

**Keywords:** wide band gap perovskite, open-circuit voltage losses, iodide, defects, high performance

## Abstract

High-performance wide-bandgap (WBG) perovskite solar cells are used as top cells in perovskite/silicon or perovskite/perovskite tandem solar cells, which possess the potential to overcome the Shockley-Queisser limitation of single-junction perovskite solar cells (PSCs). However, WBG perovskites still suffer from severe nonradiative recombination and large open-circuit voltage (Voc) losses, which restrict the improvement of PSC performance. Herein, we introduce 3,3′-diethyl-oxacarbo-cyanine iodide (DiOC_2_(3)) and multifunctional groups (C=N, C=C, C-O-C, C-N) into perovskite precursor solutions to simultaneously passivate deep level defects and reduce recombination centers. The multifunctional groups in DiOC_2_(3) coordinate with free Pb^2+^ at symmetric sites, passivating Pb vacancy defects, effectively suppressing nonradiative recombination, and maintaining considerable stability. The results reveal that the power conversion efficiency (PCE) of the 1.68 eV WBG perovskite solar cell with an inverted structure increases from 18.51% to 21.50%, and the Voc loss is only 0.487 V. The unpackaged device maintains 95% of its initial PCE after 500 h, in an N_2_ environment at 25°C.

## Introduction

Perovskite solar cells (PSCs) are one of the most promising next-generation photovoltaic technologies due to their high light absorption coefficient, tunable bandgap width, excellent low-light performance, long carrier mobility, and flexible preparation (Kim et al., 2016; Bai et al., 2019; Min et al., 2021). In just over a decade, the laboratory’s power conversion efficiency (PCE) surged from 3.8% to 26.1% (Kojima et al., 2009; Best, 2024), which is almost comparable to that of crystalline silicon solar cells. The PSCs face huge challenges in moving toward industrialization due to limitations in the Shockley-Queisser (SQ) limit and poor stability (Leijtens et al., 2018; Kim et al., 2021). Tandem solar cells can efficiently harness photon energy to overcome the SQ limit. This entails appropriate wide-bandgap (WBG) perovskites (bandgap (E.g.,) > 1.65 eV) being used as the top cell, and narrow-bandgap (NBG) perovskites (Jiang et al., 2022; Wang et al., 2022; Chen et al., 2023) or silicon (Si) (Lamanna et al., 2020; Luo et al., 2023) (E.g., ≈ 1.12 eV) being used as the bottom cell. The use of bromide, instead of partial iodide, to adjust the bandgap of WBG perovskite materials has garnered interest (Eperon et al., 2014; Saliba et al., 2018). However, WBG PSCs under continuous illumination would cause problems such as photo-induced halide segregation. Halides in perovskites demonstrate nonuniform distribution in the vertical direction; bromine and iodide distribute at the top and bottom of the perovskites (Hoke et al., 2015; Zheng et al., 2022), creating nonradiative recombination centers, resulting in a much greater open circuit voltage (Voc) loss than expected. Therefore, it is imperative to address the problem limiting the performance of WBG mixed halide PSCs to achieve low-cost and high-performance tandem solar cells (Li et al., 2023).

So far, WBG perovskites always suffer from high Voc losses (Wang et al., 2024), which hinders improving the PCE. There are two main reasons for the considerable loss of Voc: i) Deep-level defects in perovskite films become recombination centers, thus affecting the lifetime of nonequilibrium minority carriers, causing irreversible damage to the solar cells, and significantly affecting the photovoltaic performance of PSCs (Shen et al., 2024). ii) The energy level mismatch between the perovskite layer and the electron transport layer (ETL) causes electrons to cross higher potential barriers during transmission. Some carriers that cannot be crossed not only fail to produce radiative recombination but also deteriorate device performance (Gan et al., 2023). To improve the efficiency of WBG PSCs, reduce recombination, and enhance carrier extraction and mobility, different new self-assembled monolayer small molecules or additive engineering are used to suppress nonradiative recombination, adjust energy level arrangement, achieve efficient carrier extraction and transmission, and improve performance. For example, Zhao et al. designed a novel self-assembled monolayer (4- (7H dibenzo [c, g] carbazol-7-yl) phosphonic acid (4PADCB)) as a hole transport layer for PSCs by constructing a new 7H dibenzcarbazole (DCB) end group, promoting the growth of high-quality WBG perovskites, and suppressing nonradiative recombination at the interface, thereby achieving efficient hole extraction (He et al., 2023). In addition, Fang et al. added potassium antimony tartrate (APTA) to the precursor of WBG perovskite as a multifunctional additive, which regulates the dynamic growth of perovskite crystals, reduces the binding energy of lead, and improves the energy level arrangement between the absorption and electron transport layers of perovskites, effectively accelerating the extraction of charge carriers and reducing the Voc loss (Hu et al., 2023). Although introducing new self-assembled molecules can improve interface contact and reduce Voc losses, the defects of lead iodide vacancies and halogen vacancies still exist. Vacancy defects can generate deep trap energy levels, inducing nonradiative recombination, leading to carrier recombination, and causing unnecessary performance defects. Therefore, understanding the types of defects present in perovskites and leveraging the synergistic passivation effect of various functional groups can effectively reduce the trap density of states, suppress ion migration, enhance carrier mobility, and significantly improve device performance and stability.

In this study, we present a doping strategy to modify perovskite thin films by introducing 3,3′-diethyl-oxacarbo-cyanine iodide (DiOC_2_(3)) into a perovskite precursor solution. DiOC_2_(3) is composed of two benzene rings, carbon double bonds (C=C), carbon-nitrogen double bonds (C=N), and carbon-nitrogen single bonds (C-N) and ether bonds (C-O-C), exhibiting a symmetrical structure with multiple functional groups. Consequently, DiOC_2_(3) with these electron-rich groups theoretically serves as a Lewis acid and base to bind to uncoordinated Pb^2+^, while C=N and C-N, as symmetric structures, can simultaneously anchor free Pb^2+^. Furthermore, the unsaturated C=C groups would modify the energy level structure of the perovskite, reducing the potential barrier during electron and hole transport, and establishing a P-N heterojunction. This effectively enhances the carrier transfer rate, benefiting from the improvement of the built-in electric field. A more stable band structure and lower defect density significantly improve the performance of PSCs. The findings indicate that the device modified with DiOC_2_(3) achieved a higher power conversion efficiency (PCE) of 21.50%, compared to the control device with 18.51% PCE. The short-circuit current density (Jsc) also notably increased from 20.18 mA cm^-2^–22.59 mA cm^-2^, the Voc loss has been only 0.487 V, and the modified device is exhibiting enhanced humidity stability. Specifically, the unpackaged device maintains 95% of its initial photoelectric conversion efficiency after 500 h, in an N_2_ environment at 25°C.

## Results and discussions

**FIGURE 1:**
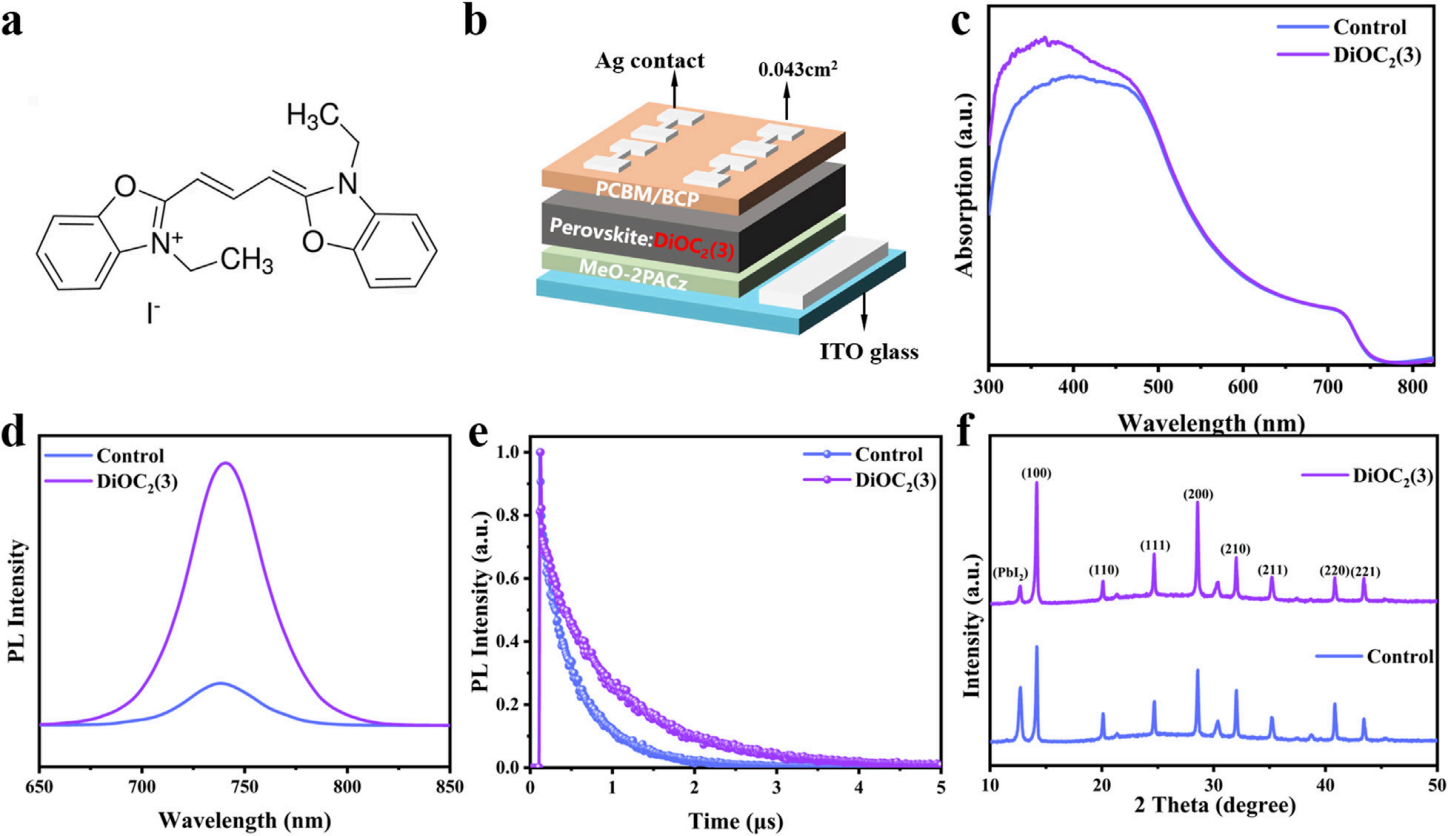
**(A)** Molecular structure of DiOC_2_(3). **(B)** Schematic diagram of the device structure with the DiOC_2_(3) introduced proportionally into the perovskite precursor solution. **(C)** The UV-vis absorption spectra of perovskite films without and with DiOC_2_(3) modification. **(D)** PL spectra and **(E)** TRPL spectra of perovskite films without and with DiOC_2_(3) modification. **(F)** XRD patterns of the perovskite films without and with DiOC_2_(3) modification.

As shown in Figure 1A, the molecular structure of DiOC_2_(3) displayed a centrally symmetric arrangement with the C=C bond at its core, flanked by C=N and C-N bonds on the left and right, respectively. Figure 1B shows the device structure diagram. DiOC_2_(3) is introduced proportionally into the perovskite precursor solution, followed by spin-coating onto the hole transport layer (HTL) and a subsequent annealing process at 100°C to facilitate crystallization. Furthermore, we fabricate and test a series of PSCs and perovskite films treated with different volume ratios of DiOC_2_(3) solution. The J-V curves of the devices are shown in Supplementary Figure S1a and the steady state photoluminescence (PL) spectra of the control film and DiOC_2_(3)-modified film are shown in Supplementary Figure S1b, Supporting Information. The PCE of the DiOC_2_(3)-modified device demonstrated a trend of first increasing and then decreasing with the increase of volume ratio in comparison with the control device, when the volume ratio of perovskite: DiOC_2_(3) is 150: 1, PSCs exhibit the best performance. In addition, the steady state PL spectra of the perovskite film show the same trend, and the PL intensity of modified perovskite film (perovskite: DiOC_2_(3) = 150: 1) is three times higher than that of the control film. Therefore, we conduct the systematic studies under the optimal volume ratio conditions. Ultraviolet-visible (UV-vis) absorption spectroscopy is performed to investigate the effect of DiOC_2_(3) on the optical properties of perovskite films. As shown in Figure 1C, the absorption spectra of DiOC_2_(3)-modified and control films are consistent with an absorption intensity of 500–800 nm, and the corresponding Tauc plots indicate the identical bandgap of 1.68 eV (Supplementary Figure S2, Supporting Information), indicating that DiOC_2_(3) does not change the bandgap of the perovskite film. Notably, within the 300–500 nm wavelength range, the absorption intensity increases following DiOC_2_(3) modification, suggesting that DiOC_2_(3) possesses additional UV spectrum absorption capabilities and effectively harnesses solar spectral energy (Dai et al., 2024). We investigate the carrier transport dynamics of DiOC_2_(3) on perovskite films by testing the steady-state PL and time-resolved photoluminescence (TRPL) of perovskite films. As shown in Figure 1D, the PL spectrum of the DiOC_2_(3)-modified perovskite film is stronger than that of the control, indicating a decrease in the defect density and an inhibition of nonradiative recombination after DiOC_2_(3) modification. The characteristic PL peaks of the DiOC_2_(3)-modified perovskite films and control film are both at 738 nm, indicating that the bandgap of the perovskite film is not changing, which is consistent with the UV-vis spectroscopy results (Guo et al., 2023; Zhang et al., 2023). Additionally, Figure 1E illustrates the TRPL outcomes for both DiOC_2_(3)-modified perovskite films and the control film, revealing that the perovskite films treated with DiOC_2_(3) demonstrate a slower decay and longer carrier lifetime. The corresponding double exponential fitting results are presented in Table 1. The fitting data is calculated according to the following Equation 1:

$$f\left(t\right)=A_{1}\exp\left(\frac{-t}{\tau_{1}}\right)+A_{2}\exp\left(\frac{-t}{\tau_{2}}\right)+B$$ (1)

**TABLE 1:** Fitting parameters of the bi-exponential decay function in TRPL spectra of 1.68 eV perovskite films without and with DiOC_2_(3) modification.

Sample (ns)	$\tau_{1}\;\left(ns\right)$	A_1_	$\tau_{2}$ (ns)	A_2_	$\tau_{ave}$ (ns)
Control	211.54	915.49	590.19	1436.80	519.79
DiOC_2_(3)	405.40	726.06	1126.03	1518.23	1020.18

Where average lifetime $\tau_{ave}=\frac{\left(A_{1}\tau_{1}^{2}+A_{2}\tau_{2}^{2}\right)}{\left(A_{1}\tau_{1}+A_{2}\tau_{2}\right)}$ (Guan et al., 2024). According to Table 1, compared to the control film, the τ_ave_ of DiOC_2_(3)-modified perovskite film increased from 519.79 to 1020.18 ns, indicating that the radiation recombination intensity of the DiOC_2_(3)-modified perovskite film is stronger, suggesting a heightened radiation recombination intensity, resulting in the inhibition of nonradiative recombination, and a noteworthy enhancement in carrier mobility (Chen et al., 2022; Chen et al., 2023). These observations imply that the DiOC_2_(3)-modified perovskite film exhibits a reduced defect density of states.

To further investigate the effect of DiOC_2_(3) on the crystallization kinetics of perovskites, X-ray diffraction patterns (XRD) of perovskite films are tested. Figure 1F shows the XRD patterns of DiOC_2_(3)-modified perovskite films and control films. The peak of PbI_2_ in the perovskite film modified with DiOC_2_(3) is significantly reduced. The peaks of (100) and (200) crystal planes are stronger, as shown in Supplementary Figure S3, Supporting Information, and the corresponding half peak width (FWHM) is reduced, indicating that DiOC_2_(3) coordinated with free Pb^2+^ through multi-site anchoring groups, forming C=N. Pb and C-N. Pb covalent bonds, thus reducing the precipitation of PbI_2_ (Cao et al., 2022; Du et al., 2022). Moreover, I^−^ ions occupy halogen vacancies, thus impeding ion migration and enhancing the crystallinity of the perovskite (Dong et al., 2023). A better crystal orientation increases the grain size, and the grain boundary resistance experienced by charge carriers during transport decreases, thereby improving the performance of perovskite thin films (Kim et al., 2019).

**FIGURE 2:**
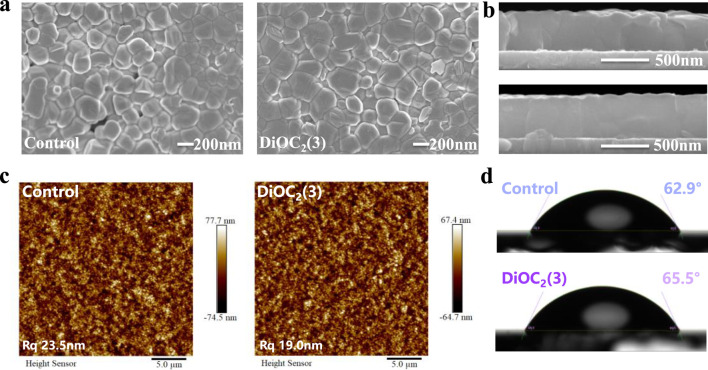
**(A)** Top-view and **(B)** Cross-sectional scanning electron microscopy (SEM) images of perovskite films without and with DiOC_2_(3) modification. **(C)** AFM images of perovskite films without and with DiOC_2_(3) modification. **(D)** Water contact angles of perovskite films without and with DiOC_2_(3) modification.

We investigate the effect of DiOC_2_(3) on the morphology of perovskite films by scanning electron microscopy (SEM) (Figures 2A,B) and atomic force microscopy (AFM) (Figure 2C). The results show that the grain size of DiOC_2_(3)-modified perovskites is increased from 160 nm to 220 nm (Supplementary Figure S4, Supporting Information), and the surface morphology becomes smoother than that of control perovskite thin films, with significantly reduced gaps and uniform grain morphology, which illustrates the increase of Jsc. In the vertical direction, as shown in Figure 2B, the grain boundaries between the grains serve as defect states, less grain boundaries in DiOC_2_(3)-modified perovskite implies suppressed defect states (Chen et al., 2023). Moreover, the AFM results in Figure 2C demonstrate that the root-mean-square (RMS) roughness of the surface of the DiOC_2_(3)-modified perovskites film is decreased from 23.5 nm to 19.0 nm. A smoother surface is conducive to the deposition of electron transport layers on the surface of the perovskite layer, resulting in tighter contact between layers and more effective electron extraction processes (Li et al., 2023). Figure 2D shows the contact angle between the DiOC_2_(3)-modified and control surfaces. The results show that the contact angle of the DiOC_2_(3)-modified surface is increased, indicating a significant improvement in the hydrophobicity of the perovskite surface (Liu et al., 2024). This is due to the ether bond (C-O-C) in the DiOC_2_(3) molecule. The difference in electronegativity between carbon and oxygen atoms makes it difficult for the ether molecule to dissolve in water, thereby improving its stability (Chen et al., 2022).

**FIGURE 3:**
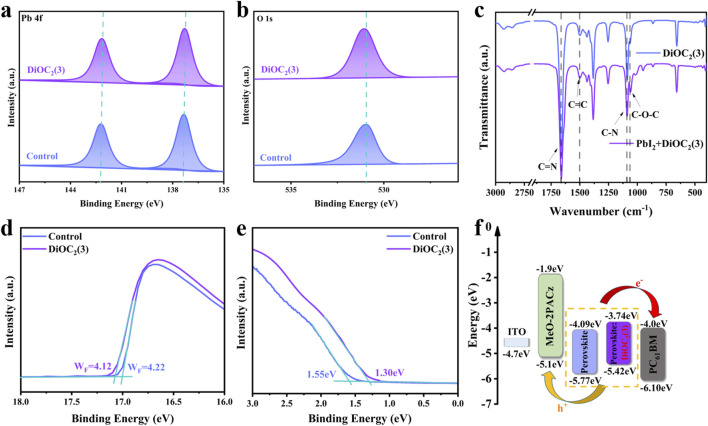
X-ray photoelectron spectrometry (XPS) spectra of **(A)** Pb 4f and **(B)** O 1s in the perovskite films without and with DiOC_2_(3) modification. The FTIR spectra of pure DiOC_2_(3) and DiOC_2_(3)/PbI_2_ are shown in panels **(C)**. UPS data of perovskite films without and with DiOC_2_(3). **(D)** Helium Iα (hν = 21.2 eV) spectra of secondary electron cut-off and **(E)** UPS spectra of the valence band (VB) region. **(F)** Schematic illustration of energy level alignment of this work.

To verify the interaction between multifunctional groups in the DiOC_2_(3) molecule and perovskite, the chemical state of the surface elements of perovskites is studied through X-ray photoelectron spectrometry (XPS). Figure 3A shows the resolution spectra of Pb 4f and O 1s. The Pb 4f peak of the perovskite film modified with DiOC_2_(3) shifts towards the direction with lower binding energy, but the shift amplitude is relatively small, indicating that the C=N and C-N groups of DiOC_2_(3) interact synergistically at symmetric sites, The C=N bond acts as a Lewis base to coordinate with Pb^2+^, as a donor for electronic units and increasing the electron cloud density around the atom (Ren et al., 2022). The C-N bond acts as a Lewis acid to coordinate with Pb^2+^, reducing the electron cloud density around the atom. However, owing to the stronger interaction of the C=N bond, Pb 4f shifts slightly towards a lower binding energy (Zhang et al., 2018). Furthermore, the C=N as an electron-rich unit, could play a Lewis acid-base role, causing the electron-rich environment of Pb^2+^ through electron donating, thereby stabilizing the structure. The peak of O 1s in Figure 3B shifts from 530.96 to 531.20 eV, indicating a strong interaction between ether bonds and the perovskite, filling the FA vacancy by binding with A-site cations. As shown in Supplementary Figure S5, Supporting Information, the peak of C 1s shifts from 284.80 to 284.93 eV, indicating that the C=C bond serves as the central site group interacting with perovskite (Jayanthi et al., 2022).

Fourier transform infrared spectroscopy (FTIR) analysis is performed on the pure solution of DiOC_2_(3) and the mixed solution of DiOC_2_(3) and PbI_2_, as shown in Figure 3C. The stretching vibration peaks of C=N and C-N groups appeared at 2,500–1,600 cm^-1^ and 1,250–1,000 cm^-1^, respectively, This proves that DiOC_2_(3) can interact well with Pb^2+^, as a Lewis acid and base to effectively passivate Pb vacancies (Cao et al., 2022). In addition, C=C and C-O-C stretching vibration peaks appeared at 1,600–1,300 cm^-1^ and 1,100–1,000 cm^-1^ (An et al., 2023; Zhang et al., 2023), indicating that the C=C bond is serving as the central site group. The C-O-C bond, as a symmetric site group, effectively stabilizes the perovskite lattice, forming a low-defect, high-crystallinity perovskite film, which is conducive to the transport of charge carriers (Shao et al., 2024). Additionally, the work function (W_F_) and valence band maximum (VBM) is measured through UV photoelectron spectroscopy (UPS) to further demonstrate the improvement of the carrier transport rate, as shown in Figures 3D–F. The W_F_ and VBM are calculated from the measured secondary electron cut-off edge and Fermi edge, respectively. Based on the VBM and bandgap (1.68 eV) of the perovskite, the minimum conduction band (CBM) of the perovskite film is determined, as shown in Figure 3D. The W_F_ modified with DiOC_2_(3) decreased from 4.22 to 4.12 eV, whereas the VBM is located at 1.30 and 1.55 eV, respectively (Figure 3E), with an upward shift in energy levels, which matches the energy levels of ETL and HTL. During electron and hole transports, the potential barrier is lowered, thereby improving the transfer rate, reducing nonradiative recombination, and decreasing the density of defect states (Stolterfoht et al., 2019; Azmi et al., 2022).

**FIGURE 4:**
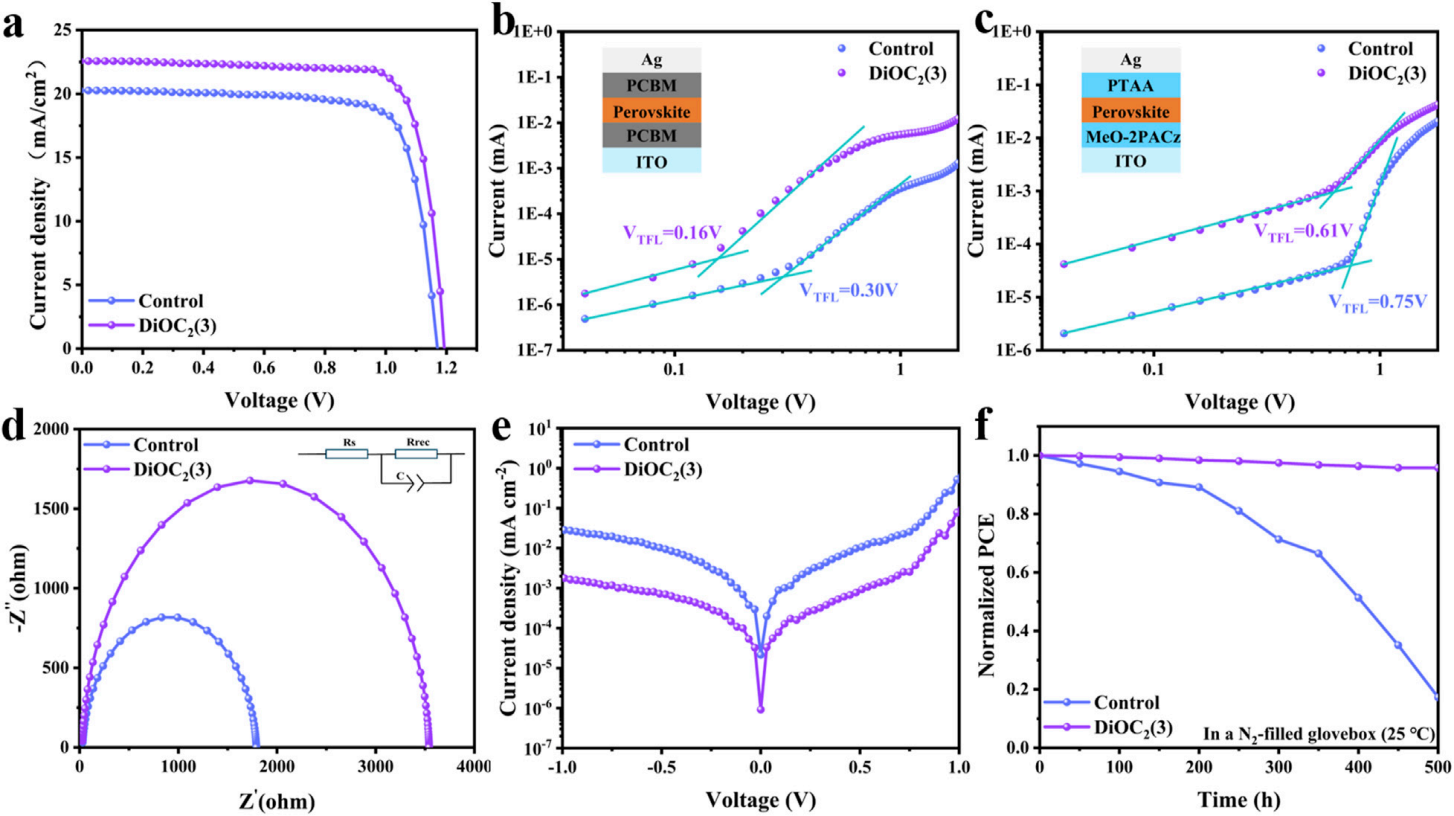
**(A)** J-V curves of the control DiOC_2_(3)-modified devices measured at 100 mW cm^-2^ irradiation. SCLC curves of the electron-only device based on **(B)** and the hole-only device based on **(C)** without and with DiOC_2_(3) modification perovskite films. The inset shows the device structure. **(D)** Nyquist plots of the two devices at a bias of 1 V in the dark. **(E)** Dark J-V curves of perovskite films without and with DiOC_2_(3) modification. **(F)** Long-term stability of the unencapsulated devices under an N_2_ atmosphere with a temperature of 25°C.

Based on this, we prepared PSCs based on ITO/MeO-2PACz/Perovskite: DiOC_2_(3)/PCBM/BCP/Ag, as shown in Figure 1B. Figure 4A shows the current density (J) - voltage (V) curve of the PSCs before and after DiOC_2_(3) modification and Table 2 shows the numerical comparison of each parameter. The control PSCs have a short-circuit current density (Jsc) of 20.18 mA cm^-2^, an open circuit voltage (Voc) of 1.170 V, and a fill factor (FF) of 0.78 and a PCE of 18.51%,. After modification with DiOC_2_(3), the PCE of the device reaches 21.50%, Jsc is 22.59 mA cm^-2^, Voc is 1.193 V (Voc loss is only 0.487 V), and FF is 0.80. As shown in Supplementary Figure S6, Supporting Information, the target device showed a higher PCE of 21.49 (21.47) % with a V_OC_ of 1.193 (1.189) V, a J_SC_ of 22.58 (22.55) mA cm^-2^, and a fill factor (FF) of 79.79 (80.13) % under reverse (forward) scan. The results show that the target PSCs display negligible hysteresis. The improvement in the device performance also proved the reduction in surface defects in DiOC_2_(3)-modified perovskite films. The external quantum efficiency (EQE) is further conducted (Supplementary Figure S7a, Supporting Information), and the results show that the DiOC_2_(3)-modified perovskite device has a higher EQE in the visible light range. The integrated Jsc of control and DiOC_2_(3)-modified PSCs are 20.52 and 22.36 mA cm^-2^. The steady-state output (SPO) efficiencies of the corresponding control and target devices at the maximum power points were 18.4% and 21.3% (Supplementary Figure S7b), respectively, which were also in line with the values determined from the J–V curves.

**TABLE 2:** The photovoltaic parameters of the DiOC_2_(3) devices.

	Jsc (mA cm^-2^)	Voc (V)	FF (%)	PCE (%)
Control	20.18	1.170	78.38	18.51
DiOC_2_(3)	22.59	1.193	79.80	21.50

To further investigate the passivation effect of DiOC_2_(3) on perovskites, we fabricate pure electron and hole devices to study the trap-state density of states using the space-charge-limited current (SCLC) method (Figures 4B,C). The density of trap-states can be calculated by formula 2:

$$N_{trap}=\frac{2{\varepsilon_{r}\varepsilon }_{o}}{eL^{2}}V_{TFL}$$ (2)

where *N*_trap_ is the density of trap-states, *ε*_r_ is the vacuum permittivity, *ε*_0_ is the relative dielectric constant for the perovskite film, *e* is the electron charge, *L* is the thickness of the perovskite layer (≈450 nm), and *V*_TFL_ is the trap-filled limit voltage (Zheng et al., 2020). Figure 4B shows the electron defect density of states, where *V*_TFL_ decreased from 0.30 V to 0.16 V, and the corresponding defect density of states decreased from 1.95 × 10^16^ cm^-3^ to 1.04 × 10^16^ cm^-3^. Figure 4C shows the density of states for hole defects, where *V*_TFL_ decreased from 0.75 V to 0.61 V, and the corresponding density of states for defects decreased from 4.88 × 10^16^ cm^-3^ to 3.97 × 10^16^ cm^-3^. The results show that the density of states for both electron and hole defects decreases, thereby increasing the mobility of electrons and holes. It is assumed that the DiOC_2_(3) modification interacts with uncoordinated Pb^2+^, occupying Pb and I vacancies. The electron impedance spectroscopy (EIS) analysis is conducted under dark conditions as shown in Figure 4D. The perovskite modified with DiOC_2_(3) exhibits a decreased in R_s_ and increased in R_rec_, further indicating the suppression of nonradiative recombination, (Zhu et al., 2022; Zhao et al., 2023). The reverse saturation current density (J_o_) is related to the defect state density and the smaller the value of J_o_, the lower the defect state density as shown in Figure 4E (Song et al., 2021; Wu et al., 2021). This further confirms that DiOC_2_(3) has a significant passivation effect on defects under the synergistic effect of multiple sites of C=N, C-N, C=C, and C-O-C bonds. Finally, we compared the humidity stability of PSCs before and after DiOC_2_(3) modification in a nitrogen glove box environment (25°C). As shown in Figure 4F, the DiOC_2_(3)-modified device maintains 95% of its initial efficiency after 500 h, whereas the control device drops down 50% of its initial efficiency after 400 h. Noteworthy that the light stability and thermal stability are shown in Supplementary Figure S8, Supporting Information. It is observed that the control devices exhibit rapid degradation after working for 200 h, while the DiOC_2_(3)-modified devices show excellent light stability and thermal stability. These results indicate that PSCs with DiOC_2_(3)-modified film possess better stability.

## Conclusion

In this study, the DiOC_2_(3) with a central symmetric structure and additional absorption in the ultraviolet band, is incorporated into the WBG perovskite precursor solution to passivate the. Pb vacancies and ion vacancy defects. The synergistic passivation effect of multiple functional group sites. could inhibit ion migration while reducing the density of defect states, improving the extraction and transport capacity of charge carriers, and achieving a lower Voc loss. The difference in electronegativity between carbon and oxygen atoms in ether bonds strongly improves the stability of the perovskite thin films. Therefore, the WBG PSCs modified with DiOC_2_(3) exhibit a PCE of 21.50%, with a Voc loss of 0.487 V. It is worth noting that the unpackaged device maintains 95% of its initial PCE in a N_2_ environment (25°C) for 500 h. Our study provides a simple and effective method for preparing high-performance, low Voc-loss, and stable PSCs.

## Experimental section

*Materials:* N, N-dimethylformamide (DMF, 99.8%), dimethyl sulfoxide (DMSO, 99.9%) were purchased from Sigma-Aldrich. Lead iodide (PbI_2_, 99.999%), lead bromide (PbBr_2_, 99.99%), Cesium iodide (CsI, 99.99%), Methylammonium bromide (MABr, 99.99%), holeblocking material 2,9-dimethyl-4,7-diphenyl-1,10-phenanthroline (BCP, 99.5%) were purchased from Xi’an Yuri Solar Co., Ltd. Formamidinium iodide (H_2_N = CHNH_2_I; FAI), [6,6]-phenyl-C61-butyric acid methyl ester (PCBM, 99.9%) was purchased from Advanced Election Technology Co., Ltd. Indium Tin Oxide (ITO coated glass, square resistance 15 Ω) is purchased from Advanced Election Technology Co., Acid (Meo-2PACz) is purchased from TCI. 3,3′-diethyl-oxacarbo-cyanine iodide (DiOC_2_(3), 99.7%) was purchased from Sigma-Aldrich. Other materials were purchased from Alfa Aesar. All salts and solvents were used as received without any further purification.

*Wide-bandgap perovskite precursor preparation:* A 1.50 M *Cs*_*0.05*_ *(FA*_*0.77*_ *MA*_*0.23*_ *)*_*0.95*_ *Pb(I*_*0.77*_ *Br*_*0.23*_ *)*_*3*_ wide-bandgap perovskite precursor was prepared by adding 19.48 mg of CsI, 188.69 mg of FAI, 36.70 mg of MABr, 532.47 mg of PbI_2_, 126.62 mg of PbBr_2_ and with 2% PbX_2_ excess, respectively(X = I or Br) in an alloyed solvent of DMF and DMSO with a volume ratio of 4:1. Then, the solutions were stirred 60 min at 60°C temperature in a N_2_ glovebox. For the perovskite with DiOC_2_(3) additive, DiOC_2_(3) was added at volume ratio 150:1 (perovskite: DiOC_2_(3)). Before preparing perovskite films, the precursor solutions were filtered through a 0.45 mm polytetrafluoroethylene (PTFE) filter.

*Fabrication of single junction wide-bandgap perovskite device:* The ITO glass substrates were sequentially cleaned with cleanser, isopropanol, deionized water, and ethyl alcohol in a sonication bath for 15 min. Then we treated the dried substrates by UV Ozone for 30 min before use. For using MeO-2PACz as a hole transportation layer, the solution in ethanol with concentration of 1.0 mg/mL was spin-coated at 5,000 rpm for 30s and annealed at 100°C for 10 min. For the WBG perovskites, 50 μL perovskite solution was uniformly dropped on the HTL by spin-coated at 1,100 rpm for 2 s, and 6,000 rpm for 35 s; In the last 10 s of spin coating, 150 μL of CB was dropped on the spinning substrates. Then, the perovskite film was immediately placed on a hot plate and annealed at 100°C for 30 min. Then, PCBM (20 mg/mL in chlorobenzene) and BCP (1 mg/ml in saturated ethanol solution) were spin-coated with spinning speeds of 1,500 rpm for 50 s and 6,000 rpm for 25 s, respectively. Lastly, 120 nm Ag was evaporated under high vacuum (<8 × 10^−4^ Pa) on the substrates to form electrodes.

*Device characterization:* Fourier transform infrared spectroscopy (FTIR) measurements were performed on a Nicolet iS50 Infrared Fourier transform microscope by Thermo-Fisher Scientific (America). J−V curves were measured using a solar simulator equipped with a 150 W xenon lamp and a Keithley 2,420 source meter. X-ray diffractometer (XRD, D8 Advance, Bruker). Atomic force microscopy (AFM) was conducted on a Bruker Dimension Icon (United States). Scanning electron microscopy (SEM) images were collected by employing a field-emission scanning electron microscope (Tescan MIRA LMS, Czech Republic). Ultraviolet−visible (UV−vis) absorption spectra were measured with a UV−vis spectrometer (UV 2600 Shimadzu, Japan). The steady-state photoluminescence (PL) and time-resolved photoluminescence (TRPL) spectra were measured using a DeltaFlex fluorescence spectrometer (HORIBA). X-ray photoelectron spectroscopy (XPS) spectra were measured using an XPS/UPS system (Thermo Scientific, Escalab 250Xi).

## Data Availability

The original contributions presented in the study are included in the article/Supplementary Material, further inquiries can be directed to the corresponding authors.
