# Supplementary material for: Additive engineering for efficient wide-bandgap perovskite solar cells with low open-circuit voltage losses
Source: Front Chem. 2024 Sep 2;12:1441057. doi: 10.3389/fchem.2024.1441057 (PMC11402806; doi:10.3389/fchem.2024.1441057)
Supplement: Supplementary file 1 [file DataSheet1.PDF]

Supporting Information

Additive engineering for efficient wide-bandgap perovskite solar cells with low open-circuit voltage losses

Xixi Yu, Huxue He, Yunuo Hui, Hua Wang, Xing Zhu, Shaoyuan Li*, Tao Zhu*
Faculty of Metallurgical and Energy Engineering
Kunming University of Science and Technology, Kunming, 650093, P. R. China

*Corresponding authors, E-mails: lsy415808550@163.com (SyL), zhutao3306@163.com (TZ)

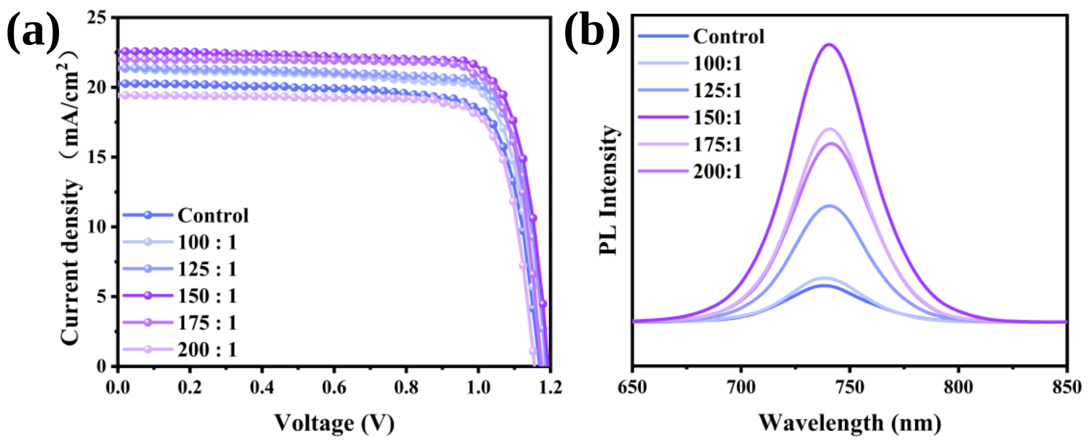

Figure S1: (a) J-V curves of the PSCs with different volume ratios of $DiOC_2(3)$-modified. (b) Steady state PL spectra of control film and $DiOC_2(3)$-modified film at different volume ratios.

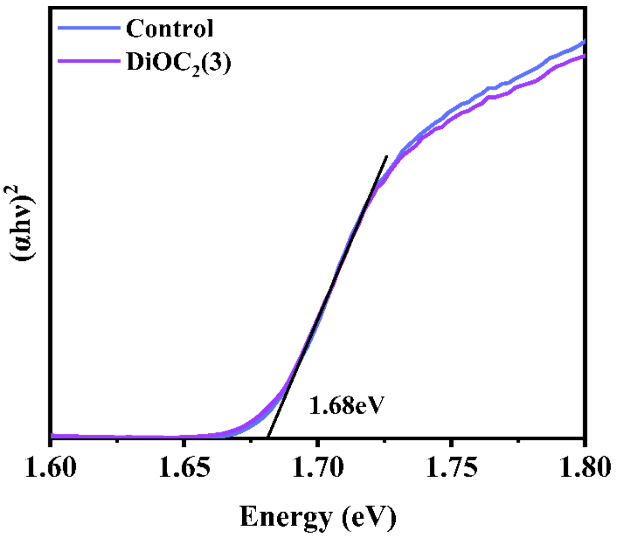

Figure S2: The Tauc plots of the perovskite films with and without $DiOC_2(3)$-modified, respectively.

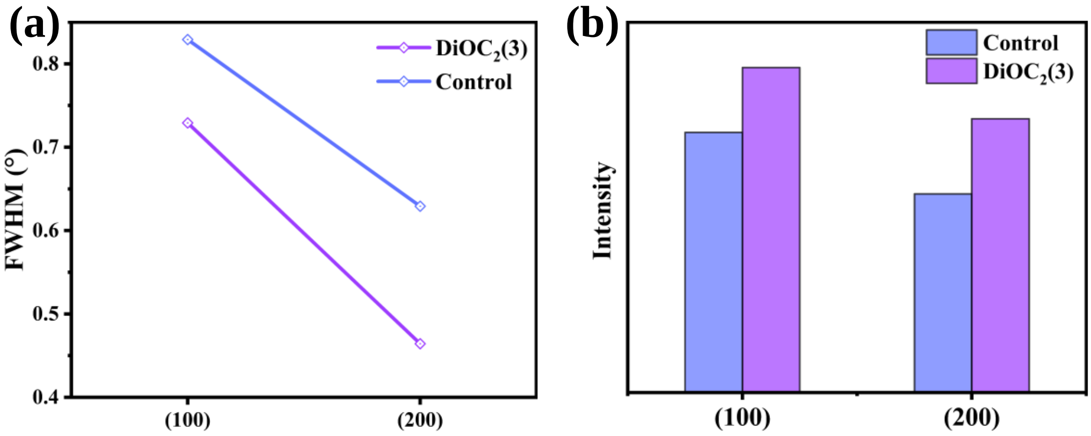

Figure S3: a) The FWHM and b) the intensity of the (100) and (200) facets for films with and without $DiOC_2(3)$-modified.

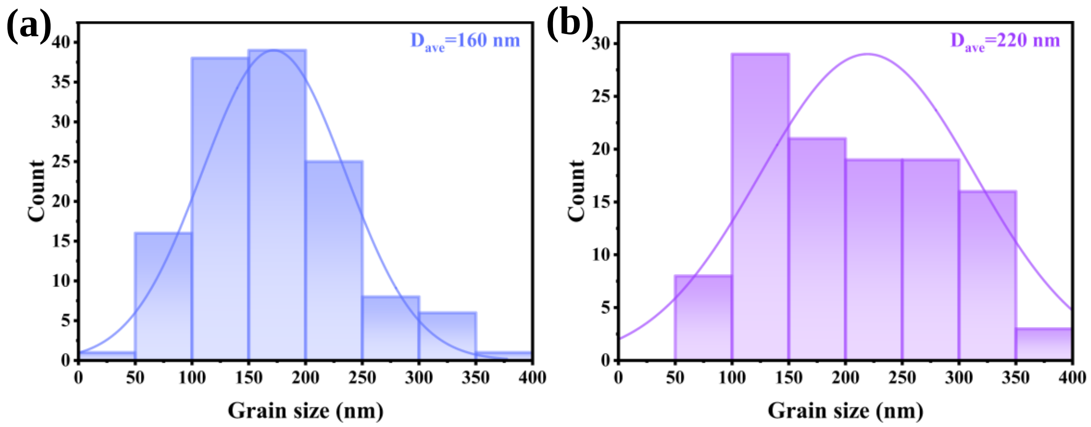

Figure S4: Histogram of perovskite grain size distributions of (a) control film and (b) $DiOC_2(3)$-modified perovskite film.

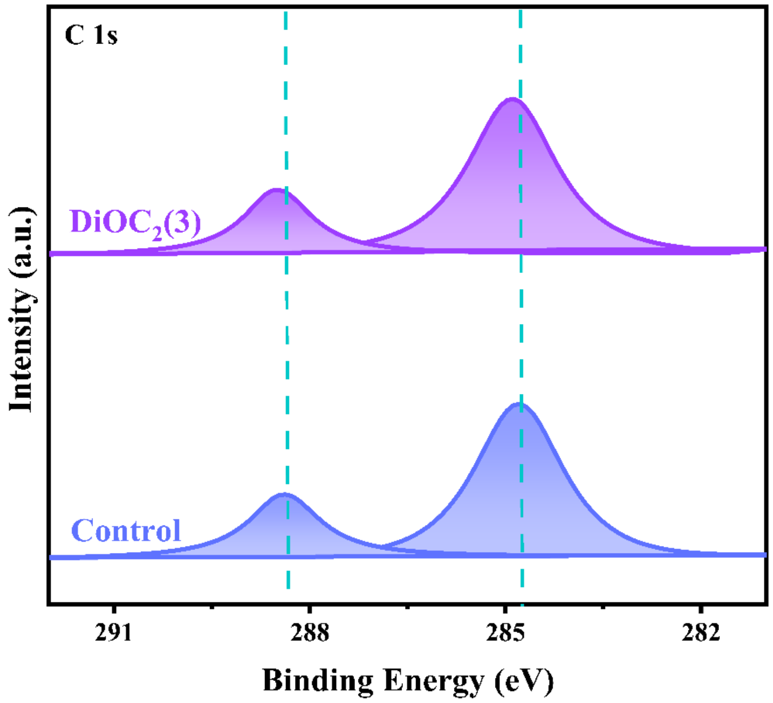

Figure S5: XPS spectra of C 1s of the corresponding films.

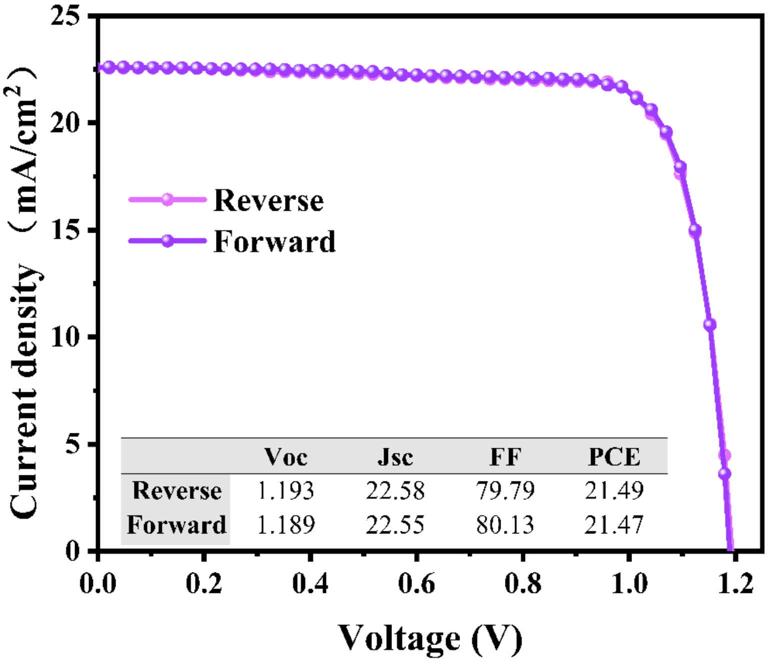

Figure S6: J–V curves of the $DiOC_2(3)$-modified PSC measured in both reverse and forward scanning directions.

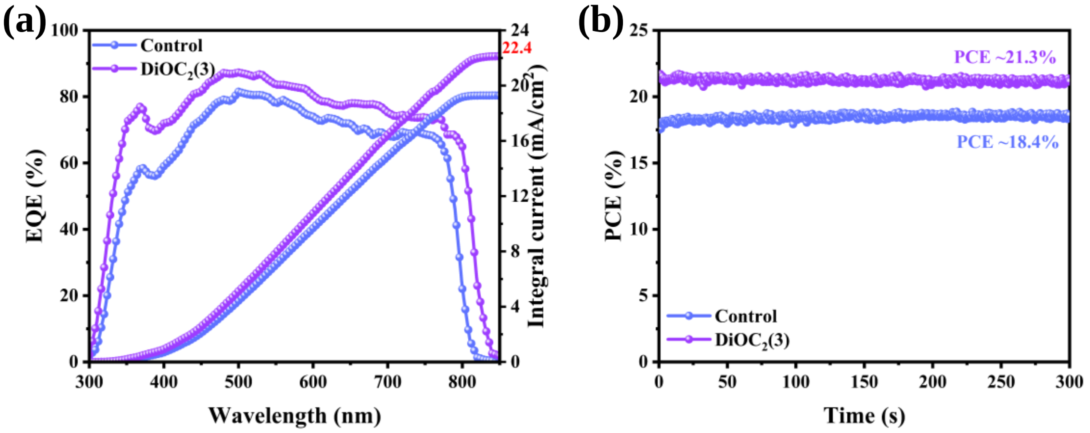

Figure S7: (a) EQE spectra and integrated photocurrent of the corresponding devices. (b) Steady-state power output of the control and DiOC$_2$(3)-modified perovskite devices for 300 s.

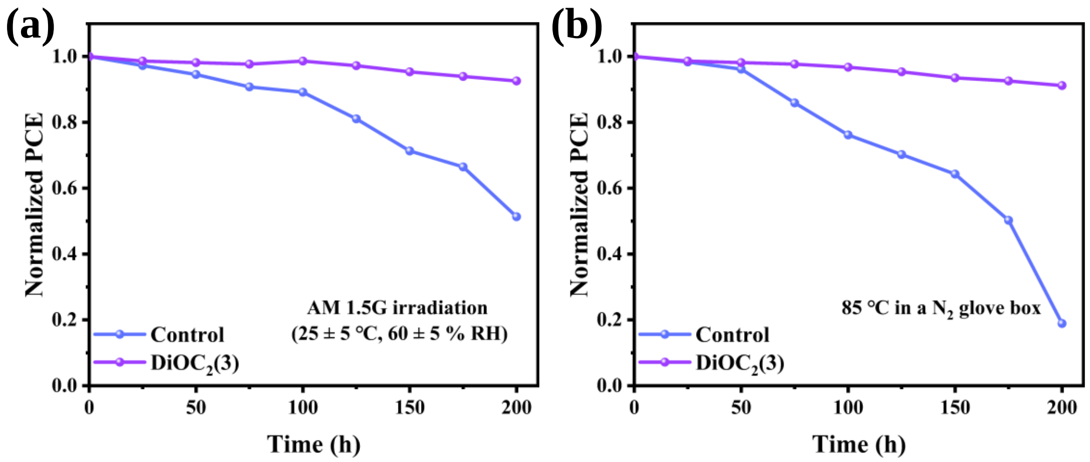

Figure S8: (a) Light stability under continuous AM 1.5G sun illumination for control and DiOC$_2$(3)-modified perovskite devices in ambient atmosphere and room temperature. (b) Thermal stability of control and DiOC$_2$(3)-modified perovskite devices without encapsulation kept at 85 °C and stored in a nitrogen-filled glove box.

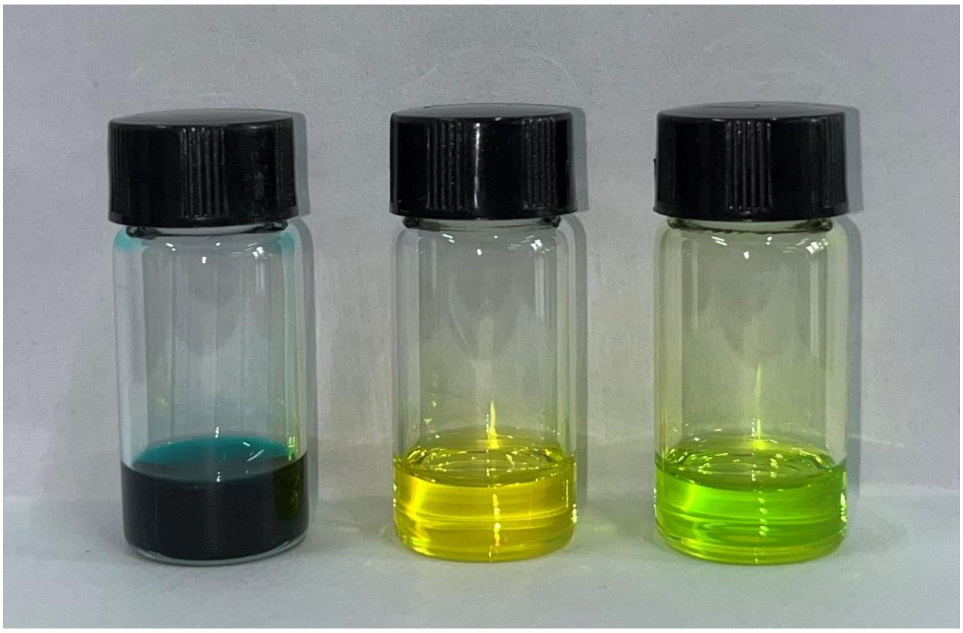

Figure S9: The three precursor fluids, from left to right, are $DiOC_2(3)$ solution, pristine perovskite solution and ternary perovskite-organic composite solution.
